# Promoting playfulness through a play-based occupational therapy intervention: A study protocol

**DOI:** 10.4102/ajod.v13i0.1415

**Published:** 2024-12-18

**Authors:** Gaby Rautenbach, Munira Hoosain, Moleen Zunza, Nicola Plastow

**Affiliations:** 1 Department of Occupational Therapy, Faculty of Medicine and Health Science, Stellenbosch University, Cape Town, South Africa; 2 Division of Epidemiology & Biostatistics, Faculty of Medicine and Health Sciences, Stellenbosch University, Cape Town, South Africa

**Keywords:** intervention, paediatrics, play, school, neurodivergence, South Africa

## Abstract

**Background:**

Play is integral to optimal childhood development. Occupational therapists often use play to improve play skills. However, when it comes to learners with autism spectrum disorder (ASD) in South Africa, there is limited research regarding play-based interventions that address the underlying motivators of play, namely, social play and playfulness.

**Objectives:**

In this study, the authors present a rationale for the development of a protocol for a study aiming to measure the impact of the Playbox Africa Intervention. This is a play-based occupational therapy intervention with the aim of enhancing the playfulness, social play and occupational performance of South African children with ASD.

**Method:**

The study will utilise a within-subject, repeated measures design and will be implemented over 16 weeks with 8–10 learners (aged 3–8 years) with ASD attending a developmental-centre environment in Johannesburg, South Africa. The playfulness and social play of the learners will be assessed using the Test of Playfulness (ToP). The modified Canadian Occupational Performance Measure (M-COPM) will be used to measure occupational performance factors.

**Results:**

Given that this protocol outlines an intervention that has not yet been implemented, there are no results to report on.

**Conclusion:**

The development of this protocol could encourage the adaption of existing play-based protocols, for children with ASD, perhaps within different settings or varying support needs.

**Contribution:**

Additionally, it could lay the foundation for future clinical trials and culturally relevant play-based interventions to be developed in the South African special needs context.

## Introduction

Play is one of the most invaluable contributions to a child’s optimal development (Jensen et al. 2021; Weisberg et al. 2016; Yogman et al. 2018; Zosh et al. 2018). While play skills can be learnt through structured or extrinsically rewarding play opportunities, authentic participation in play only occurs when a child is intrinsically motivated to play (Pyle, DeLuca & Danniels 2017; Yogman et al. 2018; Zosh et al. 2018). Play interactions, which occur naturally and are initiated and led by the child, have a significant impact on the development and learning skills as more internal joy is experienced during play engagement which a child has an intrinsic desire to be involved in (Yogman et al. 2018; Zosh et al. 2018).

One of the key elements of playfulness is the intrinsic motivation to play (Di Domenico & Ryan 2017; Masek & Stenros 2021; Muys, Rodger & Bundy 2016). Playfulness, as described by the occupational therapist, Bundy, in the Model of Playfulness, is the disposition to play in such a way that prioritises engagement over reality, expected outcomes and conventionality (Muys et al. 2016; Masek & Stenros 2021). The Test of Playfulness (ToP), derived from the Model of Playfulness, measures the playfulness elements of framing, suspension of reality, autonomy, unconventionality, open-ness, non-consequentiality and focus (Masek & Stenros 2021; Muys et al. 2016). Intrinsic motivation, however, underpins all the elements of playfulness as it drives initiation of, expansion of and reengagement in play (Masek & Stenros 2021).

According to the theory of intrinsic motivation, there are four factors that enhance the development of intrinsic motivation, namely, interest, autonomy, relatedness and competence. One of the forms of play that incorporates these promoting factors is social play, which refers to the parallel, co-operative, associative, collaborative, interactive or competitive play with other peers, playmates or adults (Borah 2021; Zheng, Janiszewski & Schreier 2023).

For learners with autism spectrum disorders (ASDs), these elements of playfulness such as framing, suspension of reality and unconventionality may be limited. Additionally, social play skills including turn-taking and non-verbal and verbal communication may be challenging (Bauminger-Zviely et al. 2020; Mukherjee 2017). Autism spectrum disorder is a neurodevelopmental disorder presenting with stereotypic, repetitive behaviours, restricted interests and difficulty interacting (Mukherjee 2017). Play-based interventions, however, as supported by the results of the studies presented below, could improve playfulness and social play for learners with ASD. Given the above-mentioned characteristics of learners with ASD, the Playbox Africa Intervention, outlined in this intervention protocol, aims to enhance the social play, playfulness and occupational performance of learners with ASD. Often within school-based occupational therapy, the focus is on enhancing learning skills and performance through play, instead of playing for play’s sake (Gretschel et al. 2022; Jensen et al. 2020; Lunga, Esterhuizen & Koen 2022).

When it comes to educators and the class environment, because of a lack of resources, practitioners, time, knowledge and skills regarding playfulness, guided play is limited and most learners only participate in free play (Gretschel et al. 2022). As much as free play is beneficial, for learners with ASD who have difficulty experiencing playfulness and social play, this type of approach to play could lead to isolation and limited opportunities to participate in joyful interactions, as there is no one facilitating the play in any way (Jensen et al. 2021; Gretschel et al. 2022). This is similarly the case at the Centre in Gauteng, which will be the study setting. For these reasons, the Playbox Africa Intervention protocol will emphasise the underlying motivators of play, playfulness and social play, by utilising individualised, sustainable playboxes to encourage joint play with the therapist and playmates.

The intervention will be a culturally relevant adaption of an existing Joint Play Playbox approach (Marwick et al. 2021) and will be based on play development principles and stages (Nijhof et al. 2018), as well as Bundy’s model of playfulness (Cordier et al. 2009). The authors of this protocol conducted a systematic review of play-based occupational therapy interventions that enhance social play and playfulness, and Marwick’s Joint Play Playboxes approach was identified as the most feasible play-based intervention, especially for low to middle income countries (LMIC) (Nada Hamadeh et al. 2022) and moderate to high support learners with ASD (Marwick et al. 2021; Rautenbach et al. 2024).

In this systematic review of 12 play-based occupational therapy interventions, the authors found that 11 of the interventions resulted in a moderate to large improvement in playfulness and social play for learners with ASD. It was also found that six key principles could lead to the success of a play-based intervention, which this protocol will follow, namely, combining free, structured and guided play during play sessions; using caregiver-guided home play to encourage engagement; combining toys of interest with novel play objects and scripts and using visuals and video modelling as tools to demonstrate playful behaviour (Rautenbach et al. 2024). Additionally, it should be considered that interventions of two or more months may be more effective than shorter intervention periods (Linstead et al. 2017). Minimally verbal learners with high support needs could respond more willingly to caregiver-rated play scales such as the modified Canadian Occupational Performance Measure (M-COPM) (Beheshti et al. 2022) in combination with observation-based assessments, such as the ToP (Fabrizi 2015; Henning et al. 2016; Kent et al. 2021).

The systematic review highlighted that most interventions focused on learners who presented with verbal communication and moderate to high intelligence quotient (IQ) scores. The research involving minimally verbal or high support ASD participants was significantly limited, as only two of the reviewed studies focused on such learners (Dionne & Martini 2011; Fabrizi 2015) and only one study was conducted in an LMIC (Anu, Sugi & Rajendran 2019). In high-income countries (HIC), for those with disabilities, specialised services such as occupational and speech therapy, physiotherapy, mobility and alternative communication devices, affordable health care and education are more easily accessible. Similarly, more play opportunities can be provided, as play spaces, play time, play objects and practitioners who are knowledgeable regarding play are accessible (Franz et al. 2017; Lunga et al. 2022; Pillay, Duncan & De Vries 2022). These opportunities, however, are significantly limited, but perhaps even more necessary in LMICs (Pillay et al. 2022).

It has been reported that, on a global scale, 95% of all learners with disabilities, including ASD, live in LMIC. The research, however, on how these children are treated, assessed and identified is scarce (Franz et al. 2017; Lunga et al. 2022; Pillay et al. 2022). Play-based, South African research focusing on ASD is also significantly limited. South Africa is an LMIC that has a population of 60.6 million people of diverse socio-economic and socio-cultural backgrounds, and vast socio-economic disparities between rich and poor (Nada Hamadeh et al. 2022; Natalie Cowling 2023). Given these characteristics, children with disabilities, including those with ASD, living in South Africa face the highest risk of not receiving quality health care and specialised education (Pillay, Duncan & De Vries 2021).

## Play-focused research in sub-Saharan Africa

Even in schools where there is some access to occupational therapy, the emphasis is often on academic performance rather than playing for plays’ sake. In 2016, Ogunyemi and Ragpot examined educators’ and caregivers’ views on work and playful learning in Nigerian and South African government-funded school contexts.

The study was not specific to learners with disabilities; yet, it was found that the creation of a play-rich environment in all sectors of childhood education in Nigeria and South Africa is a challenge. Challenges include stakeholders who are not aware of the value of play, educators who are uninformed about a play pedagogy, limited facilities, inadequate funding, policy inconsistency and poverty and diseases (Ogunyemi & Ragpot 2016).

In 2020, Jensen and colleagues conducted a cross-cultural comparison of how educators in 8 South African and 12 Canadian early childhood development classrooms guided play. Educators qualitatively reported enhanced social-emotional development of learners when play was used as a learning tool within the classroom. Suggestions as to how guided play could be incorporated into school settings were provided, including strategies such as active involvement, problem-solving and developmentally appropriate toys (Jensen et al. 2020). Lunga and colleagues (2022) echoed these findings with their play-based pedagogy for holistic development in South Africa. Through qualitative, action-based research, it was highlighted that social-emotional awareness could be improved by providing guided social play opportunities within the class environment (Lunga et al. 2022).

In a qualitative-descriptive study, addressing the promotion of play for learners with ASD, Gretschel and colleagues (2022) generated the themes acknowledging the child’s need for being in control of their play, making preferred toys available and adapting play with specific pairing with playmates, which describe the ways in which caregivers and educators promoted the play of children with ASD. They emphasised that occupational therapists should continue to collaborate with caregivers and educators to ensure that interventions focus on play, which is child directed by creating spaces that foster the child’s motivation to play and their internal locus of control (Gretschel et al. 2022).

### Play-based interventions for learners with autism spectrum disorder

Play-based interventions that improve motivation to play are imperative to developing play skills. Increased intrinsic motivation to play could lead to an increased number of playful interactions, and thus, play skills could be learnt and practised more. Consequently, this could contribute to improved overall occupational performance (Gretschel et al. 2022; Masek & Stenros 2021; Parker 2019). Francis and colleagues (2022) showed that play-based interventions can also have a positive, beneficial impact on the mental health of children with ASD. Improved mental health can lead to positive effect and increased internal motivation to play (Francis et al. 2022).

Kuhaneck, Spitzer and Bodison (2020) as well as Kent and colleagues (2020), through previous systematic reviews, have shown that play-based interventions improve play skills such as sharing and emotional awareness (Kent et al. 2020; Kuhaneck et al. 2020). Play-based interventions have also been shown to improve the social skills of learners with ASD, if underlying motivations for play are addressed through the intervention (Deniz et al. 2022; O’Keeffe & McNally 2023).

### Aim

The aim of this protocol is to develop and then determine the effect of a play-based occupational therapy intervention on the playfulness, social play and occupational performance of children with ASD.

The research question that will be answered is:

*What is the effect of a play-based occupational therapy intervention on the playfulness, social play and occupational performance of learners with ASD, within a school for differently abled learners?*

### Methodology

This clinical protocol follows the guidelines stipulated by the Standard Protocol Items: Recommendations for Interventional Trials 2013 (SPIRIT 2013) statement (Chan et al. 2013). Outlined below is the clinical protocol for the Playbox Africa Intervention, which will follow a within-subject repeated measures design. The Playbox Africa Intervention is a play-based occupational therapy intervention focused on developing the playfulness and social play of learners with ASD. An existing playbox intervention (Marwick et al. 2021) was adapted to be culturally relevant for the African context, hence the name ‘Playbox Africa’. During intervention sessions, individualised playboxes are used to encourage the learners to participate in social play with the interventionists and playmates. The playboxes include culturally relevant toys and objects, specific to the participant’s interests, along with novel play objects and scripts that are gradually introduced as the participant feels comfortable.

### Protocol version

Protocol version 03 September 2024.

This clinical protocol uses the stipulated headings for the reporting of intervention and clinical trials, as required by the SPIRIT 2013 guidelines (Chan et al. 2013).

### Funding

The Centre, which will be the study context, will not be sponsoring or funding the study. All costs related to this research study will be self-funded by the author who is the primary investigator (PI).

### Roles and responsibilities

The author and PI of this study will be responsible for training the occupational therapists (interventionists) at the Centre to implement the Playbox Africa Intervention. Training will include watching videos of the various play development stages that the intervention incorporates; role-playing the play interaction between the therapist and the learner while using the playbox to encourage playfulness and social play; providing ideas as to which toys, visuals, scripts and objects could be used for the individualised playboxes; education regarding playfulness, social play and how the play-based intervention could sustainably be introduced for moderate to high support learners with ASD and an outline of the timeline of the intervention and expectations, such as pre- and post-tests, amount and structure of sessions.

**FIGURE 1:**
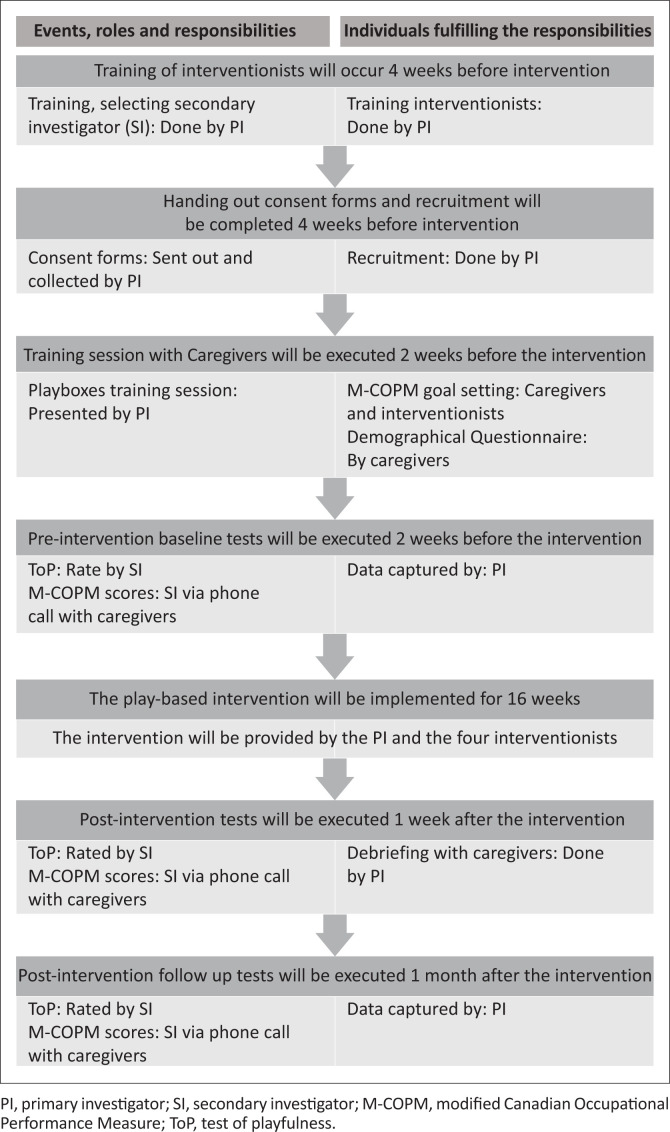
Roles and responsibilities according to intervention timeline.

A secondary investigator (SI), also an occupational therapist at the Centre, but not an interventionist, will be trained by the PI to administer the ToP to participants to reduce bias (see Figure 1). The PI and four interventionists, along with the caregivers of the participants, will be responsible for rating the M-COPM. The PI will also collect and record participant demographic information from the guardians using a questionnaire, which will be summarised as seen in Table 2. The PI will be responsible for recording the data from the ToP and M-COPM baseline tests, pre- and post-intervention tests and follow-up tests (see Figure 1). The PI will also be responsible for analysing the data, drawing up the intervention research report and disseminating the research findings.

### Objectives

The objectives of this study are:

(1) To evaluate the effect of play-based occupational therapy on the playfulness of learners with ASD using the ToP. (2) To evaluate the effect of play-based occupational therapy on the social play of learners with ASD using relevant goals pertaining to each of the learners’ activities of daily living, play, social play, learning, gross and fine motor skills identified in the M-COPM. (3) To evaluate the effect of play-based occupational therapy on the occupational performance of learners with ASD using the M-COPM.

### Hypothesis

A play-based occupational therapy intervention will enhance the playfulness, social play and occupational performance of learners with ASD in a school for differently abled learners.

## Trial design

This study will use a within-subject-repeated-measures design, which allows for the intervention group to be their own control. This design also allows for a small sample size to produce accurate results and reduces the potential effect of group contamination (Berger et al. 2021). Baseline ToP and M-COPM tests will be performed 2 weeks before the intervention period. Baseline assessments could reduce bias and add to the significance of the intervention results (Burgess, Gebski and Keech 2003; Kossyvaki & Papoudi, 2016). The outcome of baselines assessments could be compared to the intervention period results and therefore could assist in analysing whether the effect of the intervention period is significant and is truly a result of the intervention (Legoff & Sherman, 2006; Wolfberg et al., 2015).

After this 2-week baseline period, pre-intervention M-COPM and ToP assessments will be conducted the week before the intervention. Thereafter, a 16-week intervention period will take place. A 16-week intervention period was chosen for practical reasons as this period coincides with the duration of school terms. Additionally, the systematic review conducted by the authors of this protocol (Rautenbach et al. 2024) found that play-based interventions that are two or more months in length could be more effective than shorter intervention periods (Linstead et al. 2017; Rautenbach et al. 2024).

One week after the 16-week intervention period, post-intervention M-COPM and ToP assessments will be performed. The duration of 1 week was chosen given the results of the systematic review of playfulness and social play interventions, as the studies that used a pre-test post-test design measured the playfulness of the learners 1–2 weeks after the completion of the intervention. This was to ensure any improvements in play or social play as a result of the interventions were accurately and immediately noted. This duration is also practical given the length of the intervention and the structure of the school terms (Rautenbach et al. 2024). One month after the intervention, follow-up M-COPM and ToP assessments will be performed.

### Study setting

The sample intervention group will be chosen from the study population of the learners at a Centre in Johannesburg, South Africa. The Centre is registered under the Gauteng Department of Education and is classified as a ‘Centre’ for Autism. It follows the Differentiated Curriculum and Assessment Policy Statement (D-CAPS), which is aimed at Grade R to five learners with intellectual and neurodevelopmental disabilities (Department of Basic Education 2007). There are currently 70 learners with ASD, ranging from moderate to high support. The classes, which are ethnically diverse, are divided into various levels of support. The Diagnostic and Statistical Manual of Mental Disorders, version five (DSM-V), classifies ASD according to levels of support (Weitlauf et al. 2014). Level three is the highest level of support. Level two includes marked limitations in nonverbal and verbal communication skills, and level one requires lower levels of support (Weitlauf et al. 2014). The learners at the Centre predominantly fall within levels one and two, with about ten learners being level three.

### Eligibility criteria

The age band of 3–8 years for participants has been selected, as during this stage, play is central to the development and the child enjoys exploration, playing functionally and initiating games (Maree 2021; Nijhof et al. 2018; Zosh et al. 2018). The participants should have a documented diagnosis of ASD, as diagnosed by a medical doctor.

Additionally, they should have attended the Centre for at least 3 months prior to the Playbox Africa Intervention, ensuring they would have been exposed to 3 months of occupational therapy and speech and language therapy sessions at the Centre. This will ensure that each participant, even if they recently joined the Centre as a new learner, would have been exposed to the same duration and structure of therapy sessions to the extent that they will be comfortable with the style of therapy and therefore more likely to be motivated to participate in sessions without hesitation or sensory overload. New learners occasionally come from schools where there has been no exposure to therapy, which can cause initial resistance to participating in therapy.

The participants should be at a level two or three support, according to the DSM-V criteria, which is included along with the diagnosis from a medical doctor, and should have a score of 36.5 or lower on the Childhood Autism Rating Scale, version two (CARS2). The CARS2 is an effective way to supplement the DSM-V diagnosis of ASD, especially when determining the level of support each participant requires (Chu et al. 2022). A CARS2 score of 36.5 or below indicates ‘mild to moderate’ ASD, therefore relating to level two support according to the DSM-V (Weitlauf et al. 2014). This particular score range indicates that the participant demonstrates appropriate non-verbal communication skills and a certain level of joint attention and would be able to participate in a social play or play interaction (Chu et al. 2022).

Participants who present with any physical or neurological disabilities, or who are older than eight or younger than 3 years old, have been at the Centre for less than 3 months, or have a CARS score above 36.5 or are classified as ‘level one’ according to the DSM V criteria, will be excluded from the study.

### Outcome measures

Playfulness can be measured by the Test of Playfulness, version 4 (ToP), which has been validated across cultural groups and sexes and is applicable for ages 6 months to 18 years (Bundy et al. 2001; Muys et al. 2016). The ToP has been shown to be valid and reliable for children with ASD of all support levels (Harkness & Bundy 2016; Muys et al. 2016).

The systematic review conducted by the authors of this protocol found that as caregivers were integral to the implementation of play-based interventions at home, caregiver-rated play scales were used during the assessment process (Rautenbach et al. 2024). The M-COPM is an outcome measure designed to assess client outcomes in the areas of self-care, leisure and productivity. Two scores, for performance and satisfaction with performance, are obtained (Beheshti et al. 2022; Law et al. 1990). The M-COPM allows for the caregivers of a participant to collaborate with occupational therapists to set and rate goals pertaining to the participant’s occupational performance and satisfaction in the areas of leisure, self-care and productivity (Muys et al. 2016; Rodger, Braithwaite & Keen 2004).

### Outcomes

The primary outcome of this study is improved playfulness. The play-based intervention’s effect on playfulness will be analysed using the raw score of the ToP. The secondary outcome is improvement in social play, which will be measured by analysing the change in caregiver ratings of performance and satisfaction with social play goals, identified using the M-COPM. The tertiary outcome is improvement in occupational performance, which will be measured by analysing the change in caregiver ratings of performance and satisfaction for all goals identified using the M-COPM.

### Playbox Africa Occupational Therapy Intervention

This Playbox Africa Occupational Therapy Intervention outline is structured according to the SPIRIT 2013 guideline for clinical trial interventions, section 11a-d, see Online Appendix 1, Addendum A (Chan et al. 2013). The Playbox Africa is a play-based intervention to encourage the playfulness, social play and occupational performance of learners with ASD. The intervention consists of play-based, individual and group occupational therapy sessions, using joint play and a playbox individualised for the participant, a combination of free and structured play, caregivers and familiar peers who could model appropriate play and a home play programme. The intervention was developed using principles identified in a systematic review done by the authors of this study (Rautenbach et al. 2024), the Playboxes Joint Play Approach (Marwick et al. 2021) and guidelines from the play pedagogy within the South African context (Lunga et al. 2022).

### Intervention materials

Playfulness can be enhanced by focusing on the means not the end goal, non-literality, affect, which is positively displayed, and active involvement (Masek & Stenros 2021). Each participant will therefore have an individualised playbox for school, and for home, which includes preferred toys and interests. The interventionists who have an existing therapeutic relationship with the participants will guide the PI as to which play objects and visuals are appropriate for the participants. Additionally, the box will contain every-day recycled objects such as containers and straws, and objects from nature like sticks and stones. Play scripts in the form of photos or pictures will be included in the box. An individualised play box means that toys could be selected to be culturally relevant according to the culture of the participant using the box.

### Intervention procedure

**FIGURE 2:**
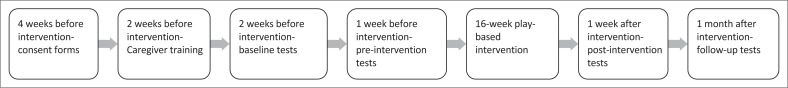
Participant timeline.

The intervention procedure is outlined in Table 1 and allows for the adaption and replication of the intervention. The Playbox Africa play-based occupational therapy intervention will focus on group, individual and home play sessions which incorporate the use of caregivers to implement the intervention within the home environment. The group sessions will emphasise social play with peers and the home sessions will work on generalising playfulness at home with siblings or caregivers, however, the use of the playbox with the learners’ unique toys of interest, will be used throughout all the sessions (see Table 1 for details). Figure 2 outlines the intervention timeline with regards to when the pre-, post- and follow-up assessments, as well as the 16-week intervention will take place.

**TABLE 1:** Details of intervention procedure.

Intervention item	Explanation and reasoning to allow for replication of the intervention
Intervention duration	16 weeks
Intervention sessions	Two weekly 35 min individual sessions and one group session led by occupational therapists (interventionists). 10 min – 15 min free play during sessions and then joint play using the playbox. Sessions will occur with participants’ usual therapists given the existing therapeutic relationship the participants have with their therapists.
Structure of sessions	A play scenario will be decided upon before the session, according to the participant’s interest, their therapy goals, sensory needs, playfulness, age and developmental stage. The structure of the intervention below will be used as a guide regarding the play development stage of the child; for example, if the participant has mastered functional play and enjoys role-play, this could be the focus, and the intervention could begin from week eight. The session will begin with the occupational therapist modelling the play combined with visuals or videos that demonstrate the desired playfulness depending on the level of development and age of the child. Toys will then gradually be taken out of the box along with participants’ preferred play items. The preferred toy encourages engagement so that more novel toys can be included in the play. The free play will be allowed to occur according to the participant’s choice and the participant’s lead will be followed during play.
Content of sessions	Based on the theory of the development of play (Lunga et al. 2022; Nijhof et al. 2018; Parker 2019), Marwick’s Joint Play Playboxes Approach will also used as a guide, along with culturally relevant adaptions according to the participants’ needs.
Group sessions	One 35 min group session led by an occupational therapist will occur per week, with two or more participants and their individualised playboxes present. Group sessions follow the same progression and structure as the individual sessions, with social play and peer interaction being the focus.
Weeks 1–3†	Functional play (e.g. using a toy car as a representation of a real car).
Weeks 4–7	Fantasy play (e.g. objects such as sticks or stones represent a real person or object).
Weeks 8–12	Role-play (e.g. participant pretends to take on the role of a vet or doctor).
Weeks 13–16	Consolidation-reduced modelling and prompting. Encouraging participants to create their own play scripts.
Grading and Tailoring	Use of M-COPM goals to guide and adapt sessions accordingly. Reducing the amount of hand over hand or verbal prompting to upgrade. Inclusion of more unfamiliar toys or playmates to upgrade. Introducing new play ideas and scripts for the participants to upgrade. Changing the environment in terms of stimuli such as noise or venue to downgrade. Increasing modelling and use of visuals to downgrade.
Home Playbox Play	Home play will occur using the individualised home playbox and will be facilitated by a caregiver who would have attended a training workshop prior to intervention. The training will be based on occupational therapy adult learning principles such as acquiring knowledge, skills and attitude. The training will be focused on the knowledge of playfulness, meaning of play and social play, and the caregivers will role-play the play situations and create an individualised home play box for the participants during the workshop. The training will be delivered by the PI in English, and three translators (isiXhosa-, isiZulu-, Sesotho-speaking) will be present for caregivers who require this. Home playbox sessions will occur for 35 min per week. If caregivers are unable to complete a play session, this will be reported and noted during the data collection phase. Caregivers will be encouraged weekly via messages from the interventionists to implement the playbox sessions. Video footage of each weekly play session will be taken by the caregiver and sent to PI via private message. Consent would have been provided for this. The videos will be used by the PI to provide suggestions on how the caregiver can facilitate and guide the play, which will then be provided to the caregiver. If caregivers change during the 16-week intervention, this will be noted, and the new caregiver will undergo the training virtually or in-person with the PI.

PI, primary investigator; M-COPM, modified Canadian Occupational Performance Measure.

† , Please see Online Appendix 1, Addendum A for more play ideas and details on how to replicate the intervention sessions.

### Methods of cultural adaptation

The Playboxes Joint Play Approach (Marwick et al. 2021) will be culturally adapted for the intervention context by using recycled and natural materials in the playboxes. Materials such as second-hand, wooden, or plastic beads, string, wire, sticks, stones and egg cartons are easily accessible for the Centre and are culturally relevant for the South African context. The type of animals chosen for the playboxes will also be culturally adapted to the culture of the participant. For example, if the participant comes from or relates to living in a farm environment, these are the types of animals that could be used. Individualised toys also involve cultural adaption as some learners’ preferred toys are relevant to their country, province or ethnic group of origin, and these will be included in the school and home playboxes. Toys could include cars in the form of Taxis or Minibuses, culturally relevant LEGO® figurines that represent African people or wooden dolls that can be dressed in traditional African garments. The language in which the intervention is conducted will be tailored to the individual in the form of visuals, which will feature written descriptions in the participants’ language of choice.

### Discontinuing the intervention

The guardians or caregivers of participants can choose to withdraw from the study at any time, for any reason; however, this will be recorded by the PI and noted during the data analysis stage. If the participant has completed more than 8 weeks of the intervention, these data will be used during data analysis, with the consideration that half of the intervention was not complete. If a participant leaves the Centre or falls ill and is not able to complete more than 8 weeks, these data will be excluded from analysis, and this will also be reported by the PI in the research report. Such a participant will no longer form part of the study sample, and their scores will not contribute to calculating the overall effect of the intervention.

### Improving adherence

Plans for participant retentionand adherence include the use of other staff members at the school, such as educators and speech and language therapists, reinforcing the concept of the play-based intervention. They will have been made aware of the intervention, its importance and value and the outcomes of the intervention. They will not provide the intervention; yet, they will motivate both the participants and guardians to remain part of the study when we meet as a multidisciplinary team for parent and caregiver feedback goal planning meetings that occur at least once per month. These practitioners will visually and verbally encourage the participants at school to attend therapy and can assist with the transition from the class to the therapy environment. Encouragement will also be provided on a weekly basis in the form of messages and communication books (in the participants’ diaries) by the manager of the school, who has approved the intervention. The PI willsimilarly encourage the caregivers to continue implementing the home intervention by calling or sending personal messages and providing support in terms of play resources and guidance.

### Prohibited interventions

The participants will receive their usual speech and language therapy, physiotherapy and extra-mural activities such as horse riding during the 16-week intervention period; however, the practitioners involved in these above-mentioned activities will have been informed of those who are participating in the Playbox Africa intervention, and the participants will be prohibited from participating in any other clinical or research trials during the intervention period. This will also be stipulated during the caregiver training and in the consent forms. If the participant begins new medication or the dosage of their medication is altered during the intervention, this will not be prohibited; yet, it will be recorded as this could affect the participant’s overall demeanour.

### Sample size

The sample size for this study will be 8–10 participants. The study conducted by Henning et al. investigating the effect of a play intervention on social play was used to establish the sample size (Henning et al. 2016). The sample size calculation indicated that an estimated sample size of *n* = 8 is required for a two-sample paired-means test. This is based on a mean difference of 2.61 (standard deviation [s.d.] = 1.64) on the ToP and a 5% significance level (α = 0.05) with 80% power (β = 0.2) to detect a significant result (Harkness & Bundy 2016; Henning et al. 2016).

**TABLE 2:** Participant characteristics.

Variables	Data to be collected	Method of collection	Data type	Measure of central tendency
Place of residence	Suburb	Questionnaire	Nominal	Mode and frequency (%)
Home language	Sepedi, Sesotho, Setswana, siSwati, Tshivenda, Xitsonga, Afrikaans, English, isiNdebele, isiXhosa, isiZulu, Other	Questionnaire	Nominal	Mode and frequency (%)
Gender	Male Female	Learner’s file	Nominal	Frequency (%)
Age	Date of Birth	Learner’s file	Ordinal	Mean
Level of support	Low Moderate	CARS2 Score	Ordinal	Median
Features of relationships	Number of caregivers at home Caregivers or helpers at home Number of siblings	Questionnaire with guardian Learner’s file	Ordinal	Mode
Time-dependent relationships	Additional care required Hospital admission- 3 months	Questionnaire with guardian Learner’s file	Nominal	Mode

### Recruitment

Convenience sampling will be used for recruitment. Four weeks before the intervention is implemented, all learners aged 3–8 years at the Centre will receive hard-copy consent forms to take home to their caregivers. The consent forms will be presented in English, IsiXhosa, isiZulu and Sesotho as these are the main home languages of the learners at the Centre; however, the form can be translated to any other language if a guardian or caregiver requires that. All participants who have been given consent will then be screened by an SI at the centre, with the Childhood Autism Rating Scale, Second Edition (CARS2) (Chu et al. 2022). This screening process forms part of the inclusion criteria, as to be eligible, the participant should present with a CARS2 score of 36.5 or below. Learners who meet the inclusion criteria are given consent, and provide verbal or written assent will be included in the sample.

### Data collection and management

Data collection will occur for the first time during the baseline period, at 2 weeks before the intervention. This is when the SI will collect the participants’ demographical information, M-COPM and the ToP baseline data. Data collection will then occur a second time during the pre-intervention test week, a third time during the post-intervention test week and a fourth time during the follow-up period, 1 month after the intervention. Data capturing throughout the study process will be done by the PI. The data will be entered by the PI into an Excel sheet located within the University Microsoft OneDrive. This can only be accessed by university staff and students using a two-step authentication process. The Excel sheet will also be encrypted with a password.

## Statistical analysis

Data will be analysed using STATA – version 17 or above (see Table VI). Data will be described through the use of categorical values, using count (percentage; %), and continuous variables will be described through the mean (standard deviation; s.d.) or median (interquartile range; IQR), depending on the range of values in the data set. Raw scores from the ToP and M-COPM will be analysed using a paired *T*-test. The significance level will be set at *p* < 0.05. The effect of the intervention will be reported as the difference in mean scores of the ToP and M-COPM pre- and post-intervention, with the corresponding 95% confidence interval. The effect of the intervention on maintenance of results will be reported as of the ToP and M-COPM pre-intervention and follow-up as well as the difference in mean scores between post-intervention and follow-up.

### Harms

Any potential harms that occur during the intervention will be reported according to the Centre’s policy. An incident report is written up, recorded and sent to the caregivers. The caregivers then assess the report along with video footage, which is available from the security cameras at the school. Caregivers will decide, along with the Centre manager, whether the incident requires action. This process is stated in the consent forms.

### Informed consent and assent

Assent from the participants will be gained before every assessment or play session. Assent will be gained verbally for verbal participants, for example ‘do you want to play today?’ and the learner will be provided with a verbal choice of ‘yes’ or ‘no’ by the therapist. Non-verbal learners will be provided with a written or visual choice of ‘yes’ or ‘no.’ It is expected that those involved in the study will be able to provide verbal assent as the eligibility criterion for participants include that the CARS2 score should not be higher than 36.5. Such a score indicates that participants will be able to understand and answer basic questions with regard to the research.

## Dissemination policy

If the intervention has a positive effect on the playfulness and social play of the participants, training will be executed by the PI for other professionals at other schools for differently abled learners in the surrounding area. If the intervention is successful, it will be adapted and incorporated into the daily programme of all the learners at the centre. The post-intervention debriefing session with caregivers and staff members at the school will also involve a presentation regarding findings of the research. The research results will be published in an appropriate peer-reviewed journal.

## Conclusion

By providing a detailed description of the Playbox Africa Intervention protocol, which will be adopted in this proposed study, occupational therapists and other health professionals can be encouraged to develop other play-based interventions within the field of health and medicine or for other learners with disabilities. The Playbox Africa Intervention could influence the way in which the daily programme or curriculum for learners with ASD is structured, so that more individualised, culturally relevant and guided play occurs within schools. Through developing the Playbox Africa protocol, the authors and interventionists noted that occupational therapists should continue to collaborate with caregivers and educators to foster opportunities for playfulness and social play. Caregivers and guardians are very knowledgeable as to their learner’s needs and play preferences, and this knowledge should be valued and used when implementing the intervention and creating the individualised playboxes. It is also important to consider the developmental age, stage and sensory needs of the learners when creating a play-based intervention.

Additionally, a successful play-based intervention should be culturally adaptable; individualised to the learners and involve guided, social and free play; and the level of support required by the leaners should be considered. Most importantly, the underlying motivators of play, playfulness and social play should be the emphasis of play-based interventions for learners with ASD.
